# Supplementary material for: Emergence and clonal expansion of a qacA-harbouring sequence type 45 lineage of methicillin-resistant Staphylococcus aureus
Source: Commun Biol. 2024 Mar 21;7:349. doi: 10.1038/s42003-024-06012-z (PMC10957945; doi:10.1038/s42003-024-06012-z)
Supplement: Supplementary file 2 — Supplementary Information [file 42003_2024_6012_MOESM2_ESM.pdf]

Supplementary Information

Supplementary Note 1: Bacterial isolates

To obtain a global representation of ST45 *S. aureus*, this study retrieved sequence data from three main sources (Supplementary Table 1). A total of 2,001 unique ST45 *S. aureus* genomes past our quality control criteria were selected for downstream analyses, and the associated metadata can be accessed in Supplementary Data 1.

Supplementary Table 1 – Demographic information of ST45 S. *aureus* sequence data used in this study

Source	Description	Quality control criteria	Data type
Staphopia	1,503 ST45 *S. aureus* genomes were sourced from Staphopia, which contains the largest collection of publicly available *S. aureus* isolated before 2017 [1].	Contig number < 200<br>MLST = 45	*de novo* genome assemblies
European Nucleotide Archive	288 ST45 *S. aureus* genomes were sourced from 'Global Epidemiology and Evolutionary History of *Staphylococcus aureus* ST45' [2].	Coverage ≥ 40<br>MLST = 45	Illumina short reads
Australian surveillance programs	To maximise diversity from different states and territories in Australia, 210 clonal complex 45 (CC45) MRSA were selected. These include:<br>• 13 isolates from the Australian and New Zealand Cooperative on Outcome in Staphylococcal Sepsis (ANZCOSS) study (BioProject PRJEB27932 & PRJEB22792) [3].<br>• 6 isolates from the Vancomycin Efficacy in Staphylococcal Sepsis in Australasia (VANESSA) study (BioProject PRJEB27932 & PRJEB22792) [4].<br>• 41 isolates from the 2015 Australian Staphylococcal Sepsis Outcome Program (ASSOP2015) [5], kindly provided by the Australian Group on Antimicrobial Resistance (AGAR)*.<br>• 39 isolates from the ASSOP2017 [6], kindly provided by AGAR*.<br>• 98 isolates from the 'Controlling Superbugs' study (BioProject PRJNA565795) that took place in the Victorian healthcare network [7].<br>• 13 isolates from Queensland surveillance of MRSA, kindly provided by the University of Queensland Centre for Clinical Research*.	Coverage ≥ 40<br>CC = 45	Illumina short reads

* Genomic data are available upon reasonable request.

Supplementary Figures

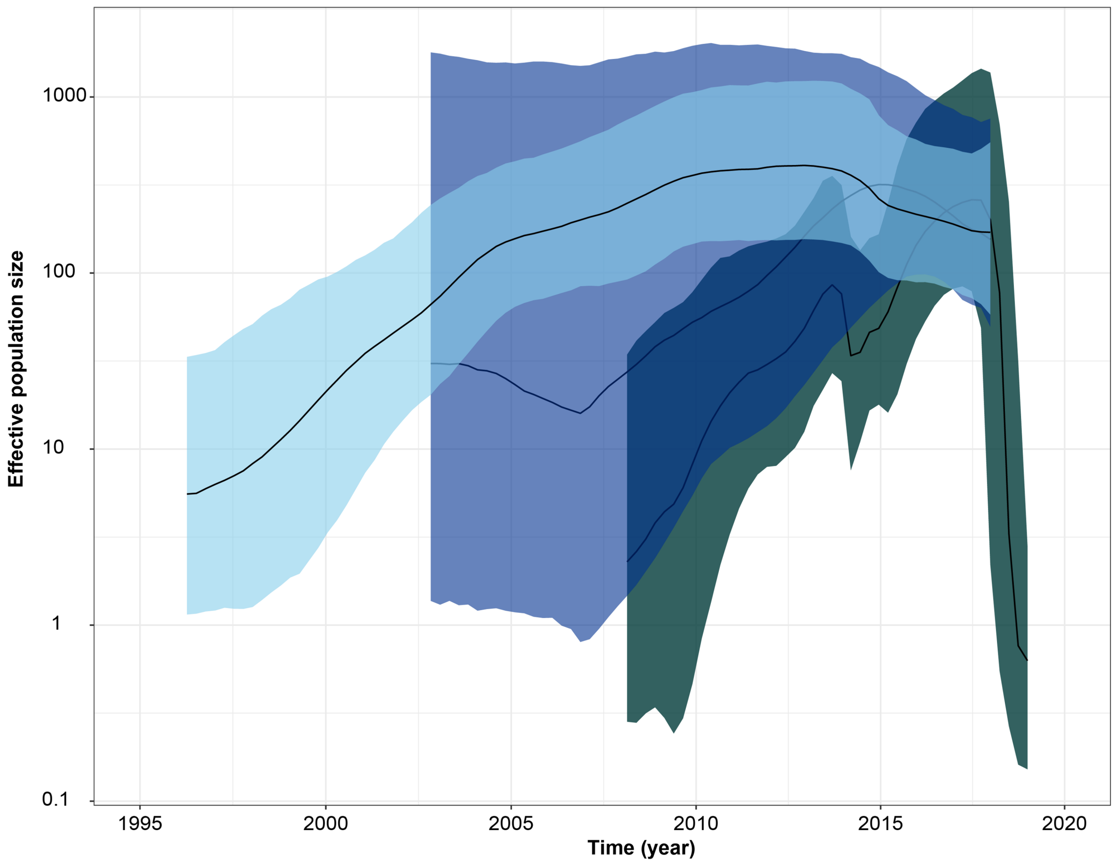

Supplementary Figure 1 – Gaussian Markov random field Bayesian skyride plot displaying effective population size (EPS) of the C2 (dark green), C4 (navy blue), and C6 (light blue) subclades. The solid black line indicates the median EPS, and the coloured boundary indicates the 95% highest posterior density (HPD).

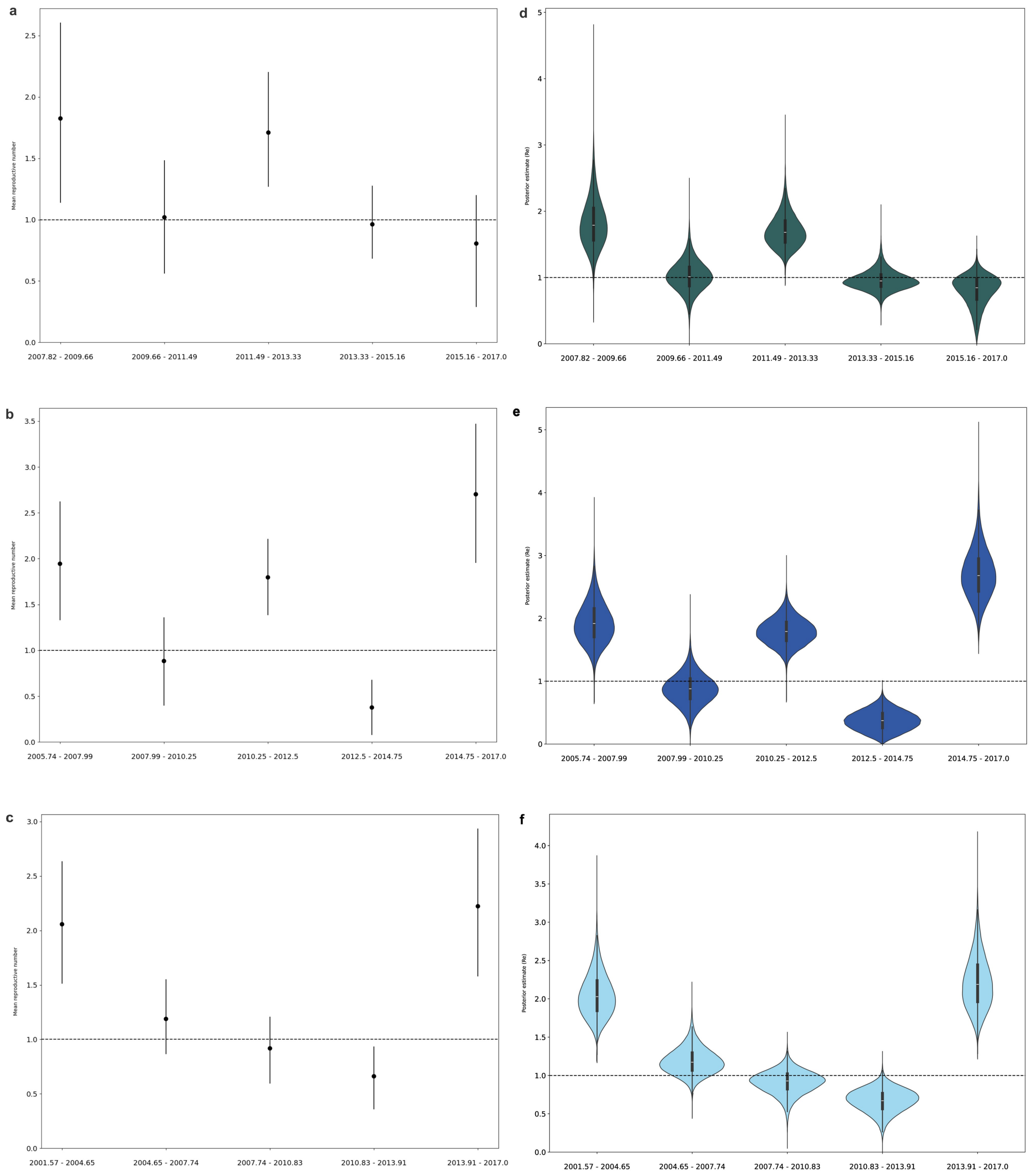

Supplementary Figure 2 – Birth death skyline estimates of the average effective reproduction number (R_e) for Australian ST45 MRSA subclades (**a**) C2B, (**b**) C4, and (**c**) C6. Confidence intervals show the 95% HPD interval in each temporal segment of the sliced Re parameter. Posterior distributions of Re for subclades (**d**) C2B, (**e**) C4, and (**f**) C6. Violin plots display the standard kernel density estimate of the Re posterior distribution for each sliced Re parameter across the estimated tree height (date ranges). Internal boxplots show the median (white horizontal bar) and interquartile range (black vertical bar); the dotted lines denote the epidemic threshold (Re = 1).

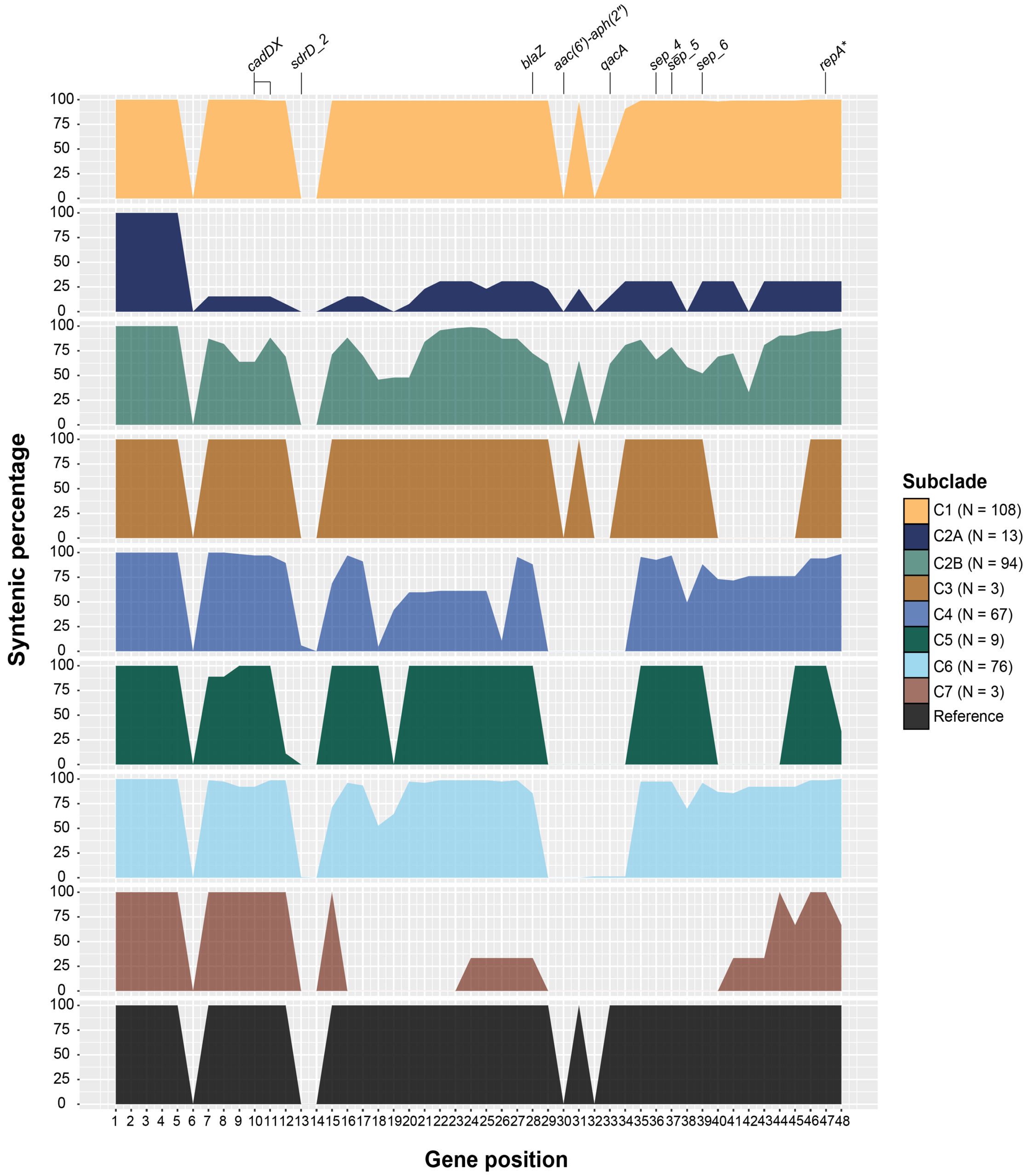

Supplementary Figure 3 – Comparison of gene synteny surrounding *qacA* between major clades in Bayesian phylogeny (Figure 2a). Genes were considered syntenic if they were physically linked on the same contig in *de novo* genome assemblies. Syntenic percentages were calculated by normalising accumulated score of gene synteny by total number of isolates in a given clade. These were then compared to the assembly of the AUSMDU00020487 genome using Illumina short-reads.

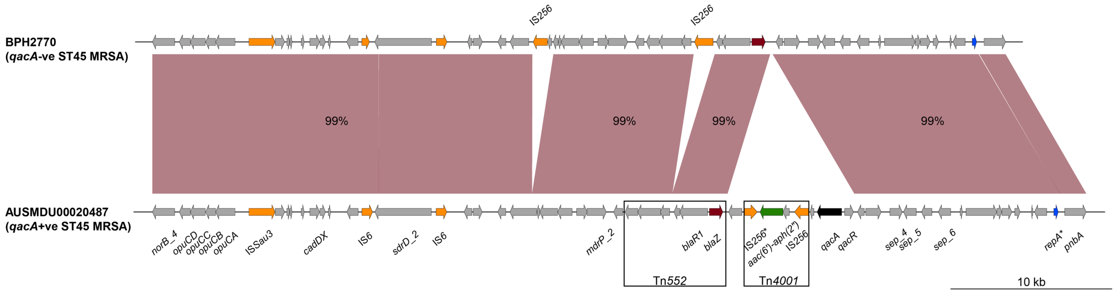

Supplementary Figure 4 – Genomic configuration of *qacA* surrounding region in AUSMDU00020487, in comparison to pF5 and pSK1 plasmids. Regions of homology (≥70%) between sequences are highlighted in the darker shades of red and blue (inversion). CDSs are shown with grey arrows. ISs (orange), plasmid replication initiator gene (blue), and AMR genes including, *blaZ* (red), *aac(6')-aph(2")* (green) and *qacA* (black) are highlighted. Asterisks indicate gene fragmentation.

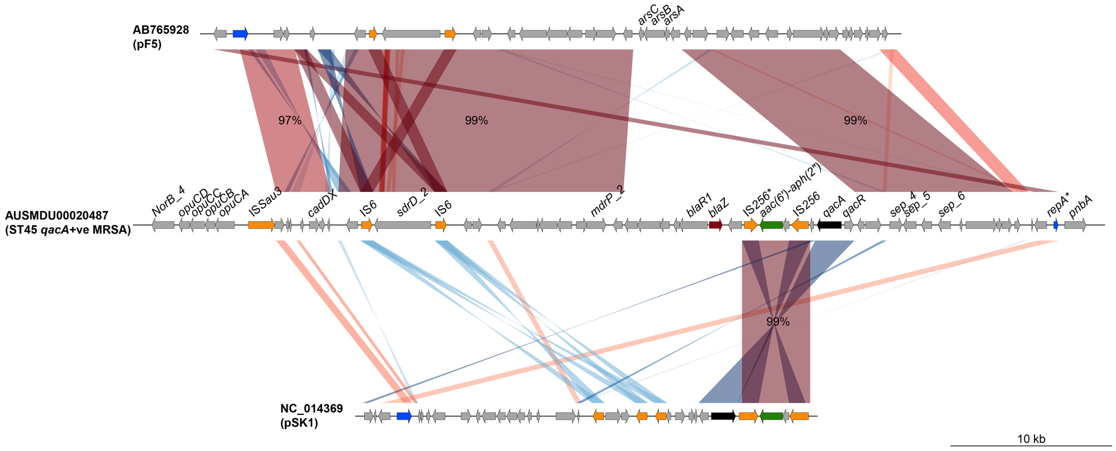

Supplementary Figure 5 – Pairwise sequence alignment between AUSMDU00020487 and BPH2770 depicting the site of *qacA* integration. Regions of homology (≥70%) between two sequences are highlighted in reddish orange. CDSs are shown with grey arrows. ISs (orange), plasmid replication initiator gene, *repA* (blue), and AMR genes including, *blaZ* (red), *aac(6')-aph(2")* (green), and *qacA* (black) are highlighted. Asterisks indicate gene fragmentation. Black rectangle boxes outline the boundary of transposons, Tn*552* and Tn*4001*.

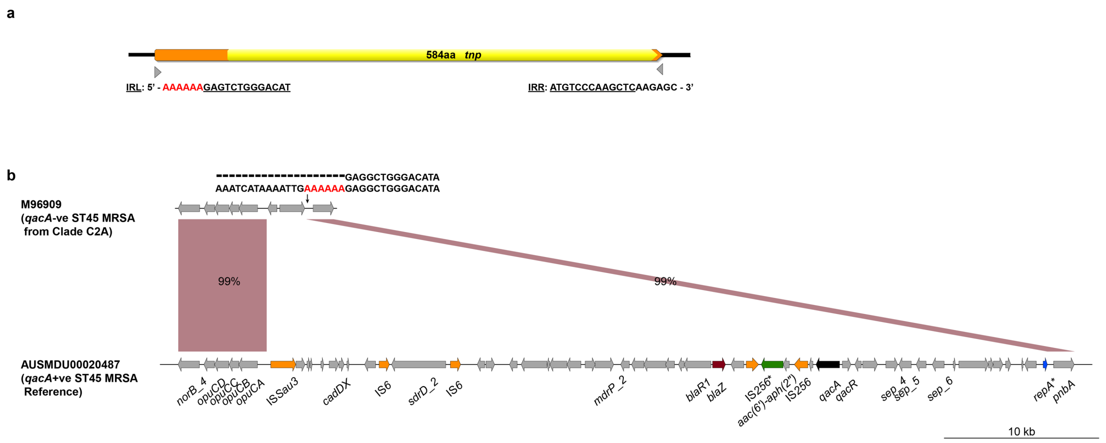

Supplementary Figure 6 – Schematic diagram illustrating IS*Sau3*-mediated excision of *qacA* surrounding region. **a** Genetic organisation of IS*Sau3*. The yellow arrow represents transpose (*tnp*) consisting of 548 amino acids. The grey triangles indicate the direction of 13 bp left and right inverted repeats (IRL and IRR) with nucleotide sequences underlined. A 6 bp homopolymer of adenine highlighted in red was identified upstream of IRL. **b** Sequence alignment between AUSMDU00020487 and M96909 showed an excision of *qacA* surrounding region likely mediated with IS*Sau3*, as the potential target direct repeat was identified in the nucleotide alignment. Regions of homology (≥99%) between two sequences are highlighted in reddish orange.

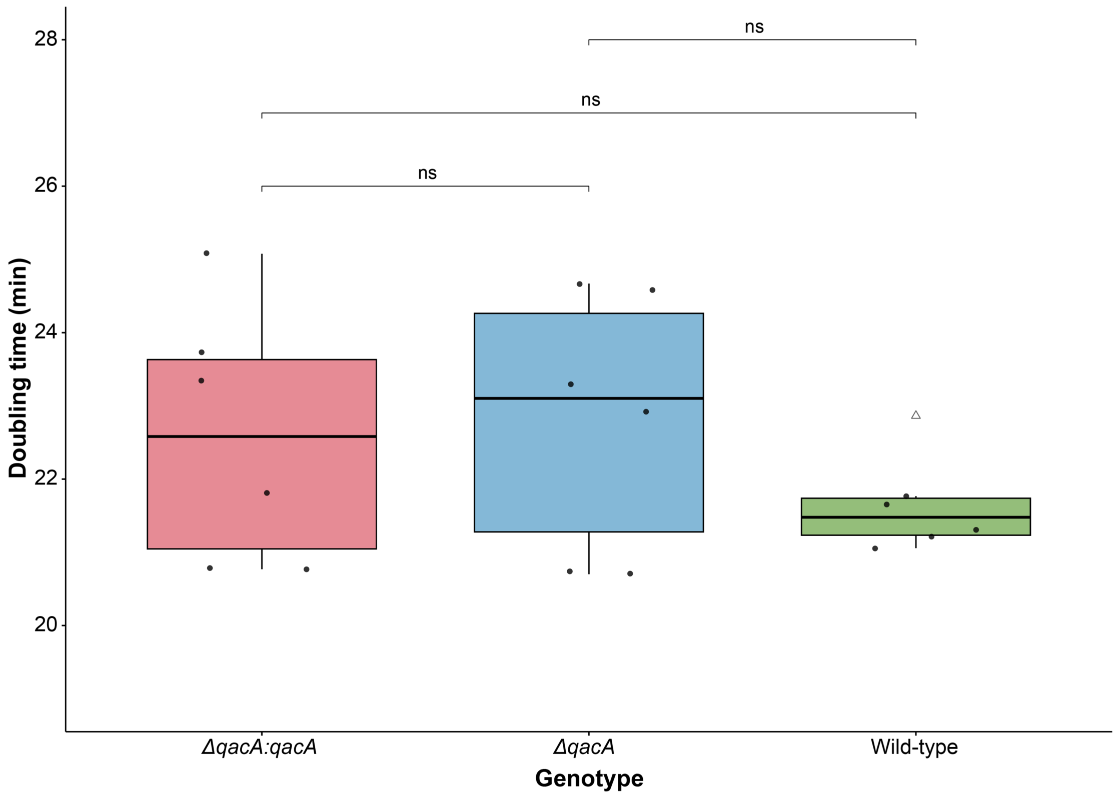

Supplementary Figure 7 – Doubling time of AUSMDU00020487 wild-type, Δ*qacA*, and Δ*qacA*:*qacA* strains. Six biological replicates denoted by the black dots were used for determining doubling times depicted with Tukey whisker boxplots. The outlier is indicated by the black triangle. No significant difference (ns, $p > 0.05$ by unpaired t test) in the mean doubling time was observed among the wild-type (mean ± standard error of the mean = 21.40 ± 0.13 min; excluding outlier), Δ*qacA* (22.82 ± 0.72 min), and Δ*qacA*:*qacA* strain (22.59 ± 0.71 min).

Supplementary References

1. Petit, R.A., 3rd and T.D. Read, *Staphylococcus Aureus Viewed from the Perspective of 40,000+ Genomes.* PeerJ, 2018. **6**: p. e5261-e5261.
2. Effelsberg, N., et al., *Global Epidemiology and Evolutionary History of Staphylococcus Aureus St45.* Journal of Clinical Microbiology, 2020. **59**(1).
3. Turnidge, J.D., et al., *Staphylococcus Aureus Bacteraemia: A Major Cause of Mortality in Australia and New Zealand.* Medical Journal of Australia, 2009. **191**(7): p. 368-373.
4. Holmes, N.E., et al., *Morbidity from in-Hospital Complications Is Greater Than Treatment Failure in Patients with Staphylococcus Aureus Bacteraemia.* BMC Infectious Diseases, 2018. **18**(1): p. 107.
5. Coombs, G.W., et al., *Australian Group on Antimicrobial Resistance (Agar) Australian Staphylococcus Aureus Sepsis Outcome Programme (Assop) Annual Report 2015.* Communicable Diseases Intelligence (2018), 2018. **42**.
6. Coombs, G., et al., *Australian Group on Antimicrobial Resistance (Agar) Australian Staphylococcus Aureus Sepsis Outcome Programme (Assop) Annual Report 2017.* Communicable Diseases Intelligence, 2019. **43**.
7. Sherry, N., et al., *Genomic Interrogation of the Burden and Transmission of Multidrug-Resistant Pathogens within and across Hospital Networks*. 2019.
