# Supplementary material for: Emergence and clonal expansion of a qacA-harbouring sequence type 45 lineage of methicillin-resistant Staphylococcus aureus
Source: Commun Biol. 2024 Mar 21;7:349. doi: 10.1038/s42003-024-06012-z (PMC10957945; doi:10.1038/s42003-024-06012-z)
Supplement: Supplementary file 3 — Description of Additional Supplementary Files [file 42003_2024_6012_MOESM3_ESM.pdf]

Description of Additional Supplementary Files

File name: Supplementary Data 1
Description: Metadata of isolates used in this study.

File name: Supplementary Data 2
Description: SNP typing of AUSMDU00020487 wild-type and mutants sequenced in this study.

File name: Supplementary Data 3
Description: Characterisation of genetic elements surrounding qacA.
